# Exploring Spiritual Concerns, Needs, and Resources in Outpatient Healthcare Facilities Serving Under-Resourced Black Patients: A Qualitative Study

**DOI:** 10.1007/s40615-024-02258-9

**Published:** 2024-12-11

**Authors:** Shena Gazaway, Kwaku Duah Oppong, Emily S. Burke, Tamara Nix-Parker, Alexia M. Torke, Shelley Varner Perez, George Fitchett, Raegan W. Durant, Rachel Wells, Marie Bakitas, Deborah Ejem

**Affiliations:** 1 School of Nursing, University of Alabama at Birmingham, 485K, 1701 University Blvd, Birmingham, AL 35294 USA; 2 School of Nursing, University of Texas at Austin, Austin, TX USA; 3 Indiana University Center for Aging Research, Regenstrief Institute, Inc., Indianapolis, IN USA; 4 Evans Center for Spiritual and Religious Values in Healthcare, Indiana University Health, Indianapolis, IN USA; 5 Department of Religion, Health and Human Values, Rush University Medical Center, Chicago, IL USA; 6 Heersink School of Medicine, University of Alabama at Birmingham, Birmingham, AL USA

**Keywords:** Spiritual care, Serious illness, Outpatient, Under-resourced, Black

## Abstract

**Background:**

Acknowledging patients’ spiritual concerns can enhance well-being and is essential to patient-centered chronic illness care. However, unmet spiritual care needs remain a major area of suffering, particularly among under-resourced populations. Limited research exists on how spiritual concerns are acknowledged and integrated into the care of chronically ill older Black patients in these settings.

**Purpose:**

This study aimed to explore the spiritual concerns and needs of chronically ill older Black patients from under-resourced areas and to identify available spiritual support resources for patients seeking healthcare through a community safety net health service.

**Methods:**

Using a qualitative descriptive design, we interviewed 13 chronically ill, older Black patients and key clinicians (physicians, nurse practitioners, allied health, and clergy). The interview focused on patients’ illness-related spiritual concerns, sources of distress, and desired spiritual support resources. Participants also reviewed the Spiritual Care and Assessment Intervention (SCAI), a spiritual care intervention, and provided feedback on its content, format, and delivery.

**Results:**

Five themes emerged from qualitative interviews: (1) spirituality is integral to seriously ill Southern patients; (2) clinicians should strive to address spiritual health in encounters; (3) socioeconomic barriers and competing demands impact priority of accessing spiritual care services; (4) spiritual care interventions should be patient-driven, compassionate, and fully integrated into medical care as a comprehensive service; and (5) participants thought SCAI was appropriate for use but should be shortened and provided in-person to increase accessibility.

**Discussion:**

Findings will inform the development and piloting of small-scale culturally responsive spiritual care intervention tailored for seriously ill older Black adults in an ambulatory care setting.

**Supplementary Information:**

The online version contains supplementary material available at 10.1007/s40615-024-02258-9.

## Introduction

Spiritual coping is an essential resource for older patients living with serious illness and is associated with better quality of life [1]. However, the spiritual needs of older individuals living with serious illness often go unmet [2]. Nearly 65% of hospitalized older adults experience some form of spiritual distress [3]. Patients who endorse higher levels of spiritual distress are more likely to report higher levels of pain [4], depression [5], anxiety [6], resting heart rate [7], and suicidal ideation [8]. Acknowledging patient and family spiritual concerns can positively affect well-being and is essential to providing patient-centered care [9]. Patients and family caregivers who believe their clinicians have sufficiently heard their spiritual concerns may be more willing to adhere to treatment recommendations, especially in the advanced illness phase [9, 10]. However, clinicians rarely address important spiritual concerns in disease-related treatment discussions [9, 11–13]. This is especially true for patients from minority group members and under-resourced populations [14].

While improving emotional and spiritual support for Black patients remains a target of quality improvement for The National Consensus of Hospice and Palliative Care Guidelines [15], little is known about how spiritual concerns are acknowledged and integrated into the care plan between patient and clinician. Nearly 80% of Black Medicare beneficiaries have a chronic and debilitating condition such as cancer, diabetes, arthritis, and cardiopulmonary diseases [16]. Proactive spiritual care early in the chronic disease experience is an essential step in addressing structural inequalities related to palliative and end-of-life care [3]. Spiritual support specialists, such as chaplains, can lead the discussion of how to bridge the gap in palliative care inequities early in the chronic disease trajectory [17].

The study aims were to define the spiritual concerns and needs of chronically ill older Black patients from under-resourced areas, ascertain available spiritual support resources from an outpatient community safety net health service affiliated with a large urban medical center, and to determine the acceptability of a chaplain-led spiritual care intervention first developed in a sample of surrogate decision-makers of patients in the ICU for use in a predominantly minority ambulatory population with low resources.

## Methods

### Theoretical Foundation

This study is informed by the Biopsychosocial-Spiritual Model of Care (BSMC) [18], which provides a comprehensive understanding of patients in their entirety. This theory is based on the belief that humans are intrinsically spiritual. Spirituality is the part of the human that seeks to be whole or healed, while religion can be viewed as one’s expression of spirituality [19]. Illness is viewed as a disruption of spiritual well-being. Serious illnesses can disturb internal biochemical processes, the mind-body connection, and spiritual well-being, including relationships with oneself, the environment, and the transcendent. As such, healing must address the whole person, including their spirituality. Spiritual care interventions for chronically ill patients experiencing spiritual distress should, therefore, aim to restore balance across biological, psychological, social, and spiritual domains.

### The Spiritual Care Assessment Intervention

**Fig. 1:**
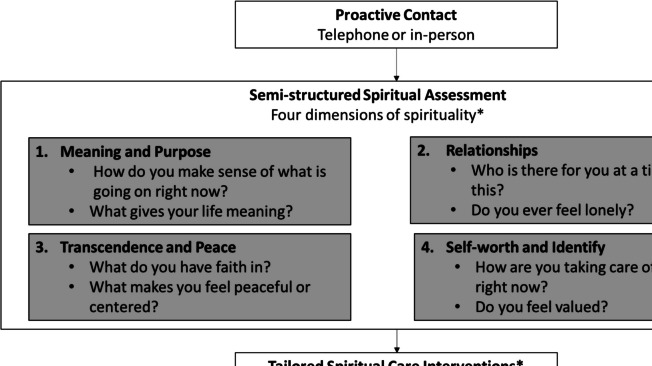
The Spiritual Care Assessment and Intervention (SCAI) framework key elements and dimension of spirituality. *Examples of questions for each spiritual dimension and spiritual care interventions are shown. The full framework is available at https://www.evanscenterindiana.org/resources/internal-resources-evans-center-resources

The Spiritual Care Assessment Intervention (SCAI) is a semi-structured chaplain-delivered intervention that consists of proactive contact, a spiritual assessment, spiritual care interventions, and documentation [20]. Developed as a spiritual care intervention for surrogate decision-makers, each component is designed to be adaptable to ever-changing inpatient contexts in complex critical care environments. The framework conceptualizes spirituality as composed of 4 broad dimensions, each of which is assessed during the intervention (Fig. 1). The chaplain delivering the intervention can select one or more questions from each dimension, based on their judgment of the individual patient. The SCAI has been pilot-tested and was effective in reducing anxiety and improving spiritual well-being in a randomized trial of ICU surrogate decision-makers [21]. It has also been piloted and tested in outpatients with cancer [22]. SCAI was developed by a racially and religiously diverse team, and the randomized trial was 2.7% Black; however, the acceptability to Black patients has not been studied. This study elicited feedback from patient and clinician participants on content, structure, appropriateness, and delivery in an ambulatory setting.

### Study Design

Following the Consolidated Criteria for Reporting Qualitative Research (COREQ) guidelines [23], we conducted a qualitative descriptive study to elicit the perspectives of chronically ill older Black patients, family caregivers (FCGs), and primary care clinicians on patients’ illness-related spiritual concerns and distress, and potential disease-related spiritual support resources for outpatient use at a community safety net health service. We also assessed the potential content, format, and delivery modality of a chaplain-led spiritual care intervention for patients experiencing spiritual distress. Study activities were conducted from January 2023 to December 2023. The Institutional Review Board at the University of Alabama at Birmingham approved this study.

### Setting and Participants

Recruitment took place at a community safety net healthcare center that is affiliated with an academic healthcare system located in Jefferson County, Alabama, and is committed to providing high-quality healthcare to all county residents, regardless of their ability to pay. This full-service ambulatory care facility includes primary and specialty care, urgent care, physical rehabilitation services, laboratory services, radiology, and a pharmacy. Patients, FCG, and primary care clinicians, including physicians, nurse practitioners, therapists, and nurses, were recruited, and based on recommendations from clinicians at the healthcare service, a community clergy member, a therapist, and a chaplain from the larger university-affiliated institution were also recruited.

Inclusion criteria (patients) included the following: (1) self-identification as Black; (2) ≥ 50 years of age; (2) having at least one of the following chronic conditions identified through electronic medical record review (EMR): heart failure, liver disease, kidney disease, cancer, chronic obstructive pulmonary disease (COPD), and human immunodeficiency virus (HIV) infection; (3) English-speaking; (4) being cognitively able to participate in semi-structured interviews. Exclusion criteria (patients) include the following: (1) Axis I psychiatric (schizophrenia, bipolar) disorder, dementia, or active substance use disorder; and (2) nursing home or assisted living facility residence.

FCGs had to be identified as “a relative, friend, or partner with whom you have a close relationship and who assists you with your medical care and who may or may not live in the same residence and who is not paid for their help.” Inclusion criteria (FCGs) included the following: (1) self-identification as a Black; (2) ≥18 years; (3) providing care to a chronically ill Black person (this person does not have to participate in a patient interview); (4) English-speaking.

### Recruitment

Patients were recruited from partnering clinicians’ clinics (nephrology, cardiovascular, and pulmonary) through an electronically generated database from a community safety net healthcare center’s EMR. Eligibility criteria (see above) were entered as screening conditions, and a comprehensive list of eligible-to-approach patients was created and shared with the study PI (D.E.) through a secured encrypted electronic file. Groups of ten patients were mailed opt-out letters that explained the study’s purpose and an information sheet detailing the study’s purpose, risks, and benefits. The letter also stated that a study team member would contact them via phone within 10 business days to answer any additional questions and provide an opt-out number if the patient desired no further contact. After 10 days, additional study details were provided during the recruitment call, including study purpose and the information sheet review; if agreeable and still eligible, verbal consent was obtained, and the interview was scheduled.

After patients were interviewed, the interviewer asked if the patient had an FCG interested in completing a similar interview. Those answering yes provided contact information, and the interviewer called the FCG to discuss the study, review the caregiver information sheet, and screen for eligibility. Upon confirming FCG eligibility and after verbal consent was obtained, the interview was scheduled or immediately took place via phone. We encountered difficulties in securing any participation from FCGs due to their limited availability and the challenges they faced in coordinating schedules, making it hard for them to find the necessary free time to participate in the interviews.

A convenience sample of clinicians whose clinics were involved in patient and FCG recruitment were invited via institutional e-mail to complete an interview. The e-mail outlined the study details and purpose and contained an information sheet and flyer as attachments. Interested clinicians e-mailed the study coordinator to make an appointment to review the information sheet. Enrolled physicians provided verbal consent, and interviews were scheduled via Zoom. Clergy were recruited based on the proximity of their churches to the healthcare facility. Team members called the clergy to invite them to participate in the study. Those who were interested provided verbal consent over the phone, and interviews were then scheduled via Zoom.

### Data Collection

During a one-time interview via an investigator-developed semi-structured interview guide based on the BSMC Framework (see Supplemental document), enrolled participants described their perspective of the patient’s illness-related spiritual concerns and distress, and potential desired spiritual support resources for outpatients at the facility.

Next, they provided feedback on potential content, format, and delivery of SCAI with Black patients with serious illness in the outpatient setting. To facilitate informed feedback, participants were mailed the intervention packet 2 weeks before their scheduled interview. Participants were provided with a $25 incentive for their participation in the interviews.

### Data Analysis

Data was analyzed by thematic analysis. As noted by Vaismoradi and colleagues [24], thematic analysis entails discovering and recognizing recurring patterns that span an entire interview or a series of interviews. It offers a qualitative, intricate, and thorough description of the data. This method also enables a rich portrayal of the phenomenon being examined, offering a detailed and contextualized comprehension [25, 26]. To begin the analytic process, two authors initially read through three interview transcripts independently to get a holistic understanding of the data. During this period, authors made field notes of short descriptions of each participant’s story about their perspective on religious/spiritual concerns and distress and the spiritual support resources available to them. Afterward, the authors began with the first cycle of coding three transcripts independently, using Microsoft Word. Next, coders resolved differences until intercoder reliability of 80% was achieved.

Using NVivo Software Version 14, we began to develop a codebook composed of code name descriptions to aid in consistently coding the rest of the interview transcripts. This process was done iteratively with periodic team meetings to discuss the coding process. Since the coding process was done independently by authors, we ensured the consolidation of all codes identified by each author by merging codes and making sure all codes were participant-direct responses that appropriately aligned with the research questions. The second coding cycle was done with the NVivo Software, aiming to identify the overall themes and categories.

## Results

### Participant Characteristics

We contacted 109 patients and 15 clinicians to participate in this study. Of these, six patients and seven clinicians agreed to participate. Reasons for non-participation for patients included passive refusal (*n* = 6), lack of interest in study (*n* = 31), deceased status (*n* = 2), being too ill to participate (*n* = 2), and no longer receiving care at the facility (*n* = 4). Among clinicians, the only reason for non-participation was passive refusal (*n* = 8). Despite multiple attempts to recruit FCG participants, none consented to participate in the study.

Thirteen interviews were conducted with patient and clinician participants from January 2023 to September 2023. Patient interviews were conducted over the phone and lasted 20–45 minutes, averaging 30 minutes. Clinician interviews were conducted over Zoom, and had a similar duration, averaging 30 minutes. All patient participants identified as Black were covered by Medicare, Medicaid, some 3^rd^ party insurance programs, or participated in the facility’s financial assistance program. They all had at least one chronic medical condition requiring regular care through services at one of the specialty clinics. The majority were female (83%), and the mean age was 57 years old. Among the clinician participants, clinician participants were 57% identified as Black and 43% as White. These clinicians represented various specialities and had provided care at the facility or a similar patient care environment for an average of 7.14 years (Table 1).

**Table 1:** Participant characteristics

Patient participants		
Age (*M = 57*)	Gender	Diagnosis
50	Male	Preferred not to disclose
59	Female	Thyroid disease Mitral valve prolapse High blood pressure
57	Female	Liver disease Kidney disease
51	Female	Kidney disease
63	Female	Rheumatoid arthritis (uses mobility aids) Kidney disease (not on dialysis) COPD
64	Female	Preferred not to disclose
Clinician participants		
Specialty	Years of service with similar population	Race
Nursing	Greater than 20 years	Black/African American
Nursing	5 years	Black/African American
Cardiology	6 years	White
Social Work	7 months	Black/African American
Social Work	10 years	White
Pulmonology	6 years	Southeast Asian
Clergy	7 years	Black/African American

### Qualitative Results

Qualitative results included four themes addressing spirituality and one theme describing general feedback about the delivery of the SCAI framework that will be useful in designing future interventions. For most patient and clinician participants, spiritual values were established during childhood. Many expressed profound faith and spiritual reverence for a higher power. Four themes emerged along with this tone of deep-seated faith and participants shared that spirituality is integral to who they were and are, and there should be an effort to address spiritual health in healthcare. They also described how socioeconomic barriers and competing demands impact patients’ perception of accessing formal spiritual care as a priority. Lastly, they reported that interventions for spiritual care should be patient-driven, compassionate, and fully integrated into medical care as a comprehensive service*.* Direct feedback was given on the current version of the SCAI, and suggestions were made for application to a predominantly minority population in ambulatory care with low resources. Participants felt that the SCAI intervention was appropriate for this population, but key improvements are needed to increase accessibility (Table 2).

**Table 2:** Themes and exemplar quotes

Theme	Exemplar quotes
1) Spirituality is integral	“Faith is such a big part of who we are.” (Clinician #5)
	“I just believe you gotta have faith in everything, and the more we put Christ in our lives or go to God, it helps. (Patient #2)
	“Hey, I’m the type that I’m gonna carry God with me wherever I go.” (Patient #6)
2) Healthcare team should strive to address spiritual health	“I think every part of a person should be an equal part of that person. My spirituality, my healthcare, my mental health care, and my socioeconomic status—all of those should be equally considered in any kind of treatment that I receive. If people have religious views that determine what they eat and don't eat, or if people view their body as a temple of the Lord, that can be a useful tool in helping somebody incorporate this new medical regimen into their life…. I think we can really see greater success rates with medical treatments if the clinicians tap into that.” (Clinician #4)
	“The doctor if they have any kind of input that they want to put into whatever the chaplain is going through or whatever kind of plan he has. I think that should be something done with him and a doctor as well as the patient and caregivers, spouse, etcetera.” (Patient #4)
3) Socioeconomic barrier and competing demands impact priority of accessing spiritual care services	“I don’t know because our patients are so—they were more about—our type of patients were more about the socioeconomic things than healthcare things: putting food on the table, paying rent, and all this other stuff.” (Clinician #1)
	Interviewer: Yes, ma’am. I was saying the spiritual care intervention that we may do with other patients at [NAME] it would be three, 30-minute phone calls. What do you think about that? Do you think that would be too long— Interviewee: That’ll be fine. Interviewer: - be good? Okay. Interviewee: Thirty minutes, I don’t—‘cause I never know when I can get that way. I’m barely making those doctors over there if the transmission’s going. We’re in a bind for too many appointments, not that I’m turning it down and don’t believe, it’s just we got a lot that we just don’t even know if we’re gonna make ‘em. Interviewer: Oh yeah, no, I understand. Okay. Interviewee: Yes, but if they could call, it would be great (Patient #3)
4) Interventions Should Be Patient-Centered, Compassionate, and Culturally Responsive	“Yeah, I think that the how often, how intense, how in-depth. I think are aspects that are probably more patient-driven…I think you get an idea for where it is in that person’s walk of life and how deeply they invest time in that space and that can inform you of how much to involve it within your care plan.” (Clinician #3)
	“That meant a lot, so I think that one of the things y’all gotta make sure is that it ain’t just a person that’s qualified for the job as far as degrees, but, man, what does he have inside of him to do this? You gotta go beyond just going, you got degrees; do you really have the care? Do you really have the patience? Do you really have the long suffering? Are you really concerned, and you want to see people spiritually be healthy about what they’re dealing with?” (Clinician #7)
	“I would definitely like to think that people should have an option to seek some type of spiritual help. Whether it’s [at the hospital] or a church or whatever, I think people should also always have an option to seek spiritual help.” (Patient #1)
5) The SCAI intervention is appropriate to use in this population, but key improvements are needed to increase accessibility	“I think the only thing that I’m gonna ever be about that is people’s reading level. That’s busy. Again, if there’s a way—I know that they’re the same questions, but if there's a way to just give an overall view, chaplain may ask you a question about something like this. Again, that’s my opinion because it might help them when they are saying…” (Clinician #6)
	“I think a lot of these terms and words are written in levels that are appropriate for less than sixth grade reading level or understanding level. I’m just going through it real quick about—yeah, none of these. These seem very simple and straightforward. I don’t think anything are gonna be too challenging for them to get…” (Patient #2)
	“Too long. The 90 minutes, is that like questions? Or you just give them time to dump off some of the problems and talk to you and pray about it? I would start with 60 minutes.” (Clinician #1)
	“I would say probably taking a more separate approach initially and then seeing how comfortable we are in bringing the group together to work through, but I think the initial approach should be separate from the care provider…” (Clinician #2)
	“Well, I think they should participate in that together. I think that’ll be best, and then that way both of ‘em know what’s goin’ on...” (Patient #1)
	“Here’s the thing about the phone call, it’s a great way to contact people but I think for the purposes of these questions, these are very intimate questions and really can do well with one-on-one in-person, I don’t think that this intervention could be delivered by phone, perhaps video. I think the video call can be helpful because you and I can read each other’s body language a little bit and it's a more intimate conversation.” (Clinician #3)

### Spirituality Is Integral

Patient and clinician participants spoke of regularly attending religious ceremonies in a church setting from childhood. All used the terms “faith” and “spirituality” interchangeably, though many spoke about faith more, especially when discussing coping techniques. Most people identified as Christian, and patient and clinician participants shared that engaging in faith-based activities in childhood was an honored family value. From a clinician perspective, this quote from Clinician #6 demonstrates the important space spirituality and faith play for patients from childhood “…it is vital down here in the South. It’s like breathing air for many people, whether Black or White, or Jewish or you name it. It’s such a function of things.” Understanding this perspective, most patients shared that they had experienced a negative life event and successfully overcome this test or obstacle through divine intervention. As stated by Clinician #7: “I know from my past to where I'm at now, it was nothing but my faith in God that pulled me out of a lot of things and turned my life around.” Many shared that they had either witnessed others (clinicians) or experienced a reconnection with faith themselves (patients) after diagnosis with a serious illness as the “distress” felt afterward triggered the desire to reconnect with their faith. Still others noted that the diagnosis created a “struggle” with their faith as they blamed lacking devotion or religious participation on their poor health. Yet, even within this context of distress and struggle, Patient #5’s quote describes what faith brings within the context of serious illness, pain, and diminishing mobility:

I thank the Lord for a lot of things. I’m still here. I’m able do what, get my cane, walk through my house and walk back in the kitchen. I can sit down at my table. I just scoot to the cabinet, to the Frigidaire and get what I need and I put everything on the table. I go in my kitchen drawer and get my silverware, and I just go ahead and get my food and stuff together. Then I just take it and sit it on the stove and dish from there. I get up and try to sweep my porch off. Then I start feeling myself get tired. I sit back down, and I just rest a little while. That’s the way I get everything together. The Lord brought me this far. I'm going to keep on, just take my time.

### Healthcare Teams Should Strive to Address Spiritual Health

Faith or belief in a higher power was discussed as a means for patients and families to cope. From a clinician’s standpoint, faith provided patients with a better outlook, endurance, and resilience. Clinicians described these patients as “…being in serious shape, and they're near the end of life, and they're bringing up phrases about God and healing and powers, and he knows the path and whatever his will is.” (Clinician #6)*.* These types of discussions described why patient participants felt the healthcare team should routinely address spiritual health.

Clinicians also shared that even in advancing illness, so few Black patients had advanced directives that they believed that spiritual care could be an avenue for discussing faith-based reasons and values for understanding the importance of having advance care planning discussions.

I might be completely wrong on this, but I would say less than a quarter or 10 percent might have an advanced directive or living will. I don’t think it’s any of ‘em. A spiritual care can be vital to that in completing that documentation with them because they’re gonna understand the values part of things and be an easy segue into the advanced care planning discussions. (Clinician #6)

However, clinicians also describe their discomfort with discussing spirituality with patients when they did not feel like they had the proper training to do so. This same clinician said, “Judging from the literature and seeing that a lot of people have spiritual care needs that are unaddressed, it probably should be raised up more than it is, and especially in our neck of the woods in the Deep South. I think a lot of people have those needs. First, I’d like to define what that [spirituality] actually means…I don’t know how to clarify that.” (Clinician #6)

Patients expressed a tangible desire to have conversations about their spiritual health because this aspect of their health was important to them. Patients discussed the importance of prayer, “I mean as far as me getting better, I hope and pray that they come up with a cure for all this stuff, especially the kidney disease, because when I found out, I was like, okay, what am I gonna do?” (Patient #4); while clinicians recalled having patients and families ask if they would pray with them, “‘You’re part of the care team, but you’ll get the power to fix my loved one,’ “that kind of thing, and praying for us” (Clinician #6). Clinician #7 shared a perspective regarding the inclusion of spirituality in care: “I think that to make this effective, you gotta have the person that’s asking the questions gonna have to do it in a way where he just ain’t reading off a list, but there’s some care that’s coming with it, that they don’t just feel like this person’s just asking me questions, but they’re legitimately concerned about what I’m dealing with,” which supports some patient sentiments that connecting can be transformative.

### Socioeconomic Barriers and Competing Demands Impact Priority of Accessing Spiritual Care Services

Participants, especially clinicians, discussed that because under-resourced populations experience barriers to basic healthcare in general, their access to services like spiritual or palliative care is also lacking. For context, the discussion highlighted the fragmented nature of essential care that leads to those with low resources experiencing longer wait times for specialty care, more emergency room visits for worsening symptoms, and thus increased suffering within the illness experience. Further discussion revealed that despite deep ties to faith and spirituality, those experiencing economic barriers that impact healthcare have multiple competing demands, and most will choose to prioritize maintaining the welfare of their homes and securing food rather than paying for healthcare. Often, when patients do decide to seek healthcare, they “…will not always speak up…” about spiritual care and well-being as it is not viewed as the priority in the moment.

### Interventions Should Be Patient-Centered, Compassionate, and Culturally Responsive

Patients and clinicians suggest that patients should be able to decide if they want to engage in spiritual health interventions, and every patient should be presented with the choice. Any intervention should allow patients to discuss their faith practices with the facilitator; the intervention should be integrated into the care the patient is already receiving, and multiple healthcare team members, including doctors, should be involved in the process. Participants also recommended including caregivers and spouses as a part of the intervention process to convey the importance of family and social network bonds. Clinicians recommended having integrated approaches to distress care to reduce barriers to palliative care access, thereby potentially reducing burden and distress for under-resourced populations.

Clinicians and patients emphasized that interventions should reflect patients’ cultural values, religious traditions, and personal preferences. For instance, understanding the nuances of a patient’s faith practice or how spirituality is expressed in their cultural context is critical for ensuring that interventions are respectful and meaningful. As one patient explained, “[The program] should work well once you talk with the patient and they give you information back to you” (Patient #5). This underscores the need for two-way communication to tailor interventions to individual needs.

Clinicians also highlighted the importance of assessing a patient’s spiritual journey to determine how deeply spiritual care should be integrated into their treatment. One clinician noted, “I think you get an idea for where it is in that person’s walk of life and how deeply they invest time in that space and that can inform you of how much to involve it within your care plan” (Clinician #3). Facilitators should be prepared to adapt their approach to different cultural worldviews, including those shaped by religious, spiritual, or familial influences. As another clinician pointed out, “[B]efore you get to [the program], you’re gonna have to ask the question of what a person believes in because you don’t want a Jehovah’s Witness in there with a person that’s a Christian or send a Christian in there with a person that’s [a] Jehovah’s Witness” (Clinician #7). This highlights the need for cultural sensitivity and thoughtful pairing of facilitators with patients.

Opinions about the right clinician to provide the intervention varied; however, a clergy member came up most often. However, the majority discussed that regardless of title, it needed to be a person interested in caring for people who could do so respectfully. Patients debated these traits more: *someone who wants to do the job, wants people to be spiritually healthy, and would be comfortable talking about sometimes uncomfortable things – such as bad family dynamics or spiritual distress due to life circumstances*. Some clinicians felt that to ensure a systematic process, the encounter should begin with a set of the same questions to be asked of everyone, and then patients and families can be provided the room to “…speak their problems out loud and have someone hear them” (Clinician #3). Overall, the idea of the intervention is to provide space for patients to share their faith and spirituality, if preferred, to support and cope throughout the serious illness experience.

### The SCAI Intervention Is Appropriate to Use in This Population, But Key Improvements Are Needed to Increase Accessibility

Participants provided their perspectives on SCAI, incorporating reflection on the current design and suggestions for implementation in a predominantly minority ambulatory care population with low resources. When offered the intervention as a handout, participants provided feedback on the general literacy level considerations, commenting on potential participants’ ability to comprehend the program. Clinician #6 shared, “I think the only thing that I'm gonna ever be concerned about is people's reading level.” This sentiment was shared by others, highlighting the need for the SCAI to consider patients’ varying levels of literacy and advocate for resources that cater to all levels of health literacy, ensuring no patient is excluded due to complex medical jargon. Participants, however, felt that SCAI was written in an accessible manner for those with low literacy levels, “…I think a lot of these terms and words are written in levels that are appropriate for less than sixth grade reading level…These seem very simple and straightforward. I don't think anything are gonna be too challenging for them…” (Patient #2).

Intervention length was discussed as a concern because SCAI is currently designed to be implemented over 90 minutes. The overall recommendation was to have the ability to adjust intervention duration based on patient’s condition, “Intervention length is too long. The 90 minutes is that like questions? Or do you just give them time to dump off some of the problems and talk to you and pray about it? I would start with 60 minutes” (Clinician #1). This feedback implies that a shorter, more focused session might be more effective in the ambulatory setting.

Participants’ opinions varied regarding the inclusion of family caregivers in SCAI. A patient shared, “Well, I think they should participate…together. I think that’ll be the best, and then that way both of ‘em know what’s goin’ on ‘cause if one gets to participate, and then when that one…they don’t know what’s goin’ on…” (Patient #6). This was echoed by multiple clinician participants who shared that the inclusion of family would help with understanding, support, and facilitate technology as needed. However, some clinician participants spoke about conducting the initial session in a private space with the patient before including family, “I would say probably taking a more separate approach initially” (Clinician #2).

The delivery of SCAI was discussed last, with the consensus being that SCAI should be an in-person interaction rather than implemented via telehealth modalities due to the sensitive and personal nature of the intervention. “I think that for the purpose of these questions, these are very intimate questions and really can do well with one-on-one in-person” (Clinician #3). In the discussion, while phone delivery was discussed, none of the participants desired SCAI to be phone-based as they thought this method would be ineffective and lack the personal presence required: “…I think it would take a very intimate conversation that I don't think can be delivered by phone as effectively” (Clinician #3).

## Discussion

This study sought to identify the spiritual needs and concerns of chronically ill older Black patients from under-resourced areas, to evaluate the available spiritual support resources at an outpatient community facility associated with a large urban medical center, and to assess the acceptability of a chaplain-led spiritual care intervention for use in a predominately minority ambulatory population with low resources. The findings of this study highlight the profound importance of spirituality in the lives of chronically ill older Black patients from under-resourced areas. Five key themes emerged: the integral role of spirituality, the socioeconomic barriers and competing demands affecting accepting formal spiritual care services, the prioritization of spiritual health, the necessity of patient-driven, compassionate interventions, and the SCAI is generally appropriate. However, some modifications should be made to make it more accessible. These themes underscore the need for healthcare facilities to incorporate spiritual care as a core service, particularly in Black communities where spiritual and religious beliefs are a central component of identity and coping mechanisms. This is consistent with studies that emphasized the existence of close links between spiritual well-being and overall health and quality of life of the Black older adult population [27–29]. This connection suggests that addressing spiritual concerns can improve patient outcomes, such as enhanced emotional health, better coping strategies, and increased satisfaction with healthcare services [30, 31].

First, the integral role of spirituality for the participants reflects a deep-seated faith and spiritual reverence. This finding aligns with previous research indicating that spirituality and religion are pivotal in Black communities, providing comfort, strength, and a sense of purpose and identity [32–34]. The participants in this study emphasized that spirituality is an essential aspect of who they are and how they experience serious illness, suggesting that healthcare providers must acknowledge and integrate this dimension into their care practices. This can be done as simply as by being open to discussing spirituality if the patient initiates the conversation during a clinical encounter [14]. Doing so can create a more holistic approach that respects and supports the whole person. Considering this, clinician participants in this study noted that few Black patients had advanced directives. They believed that spiritual care could serve as a valuable approach to discussing faith-based reasons and values, emphasizing the importance of advanced care planning discussions.

Secondly, the participants’ descriptions of the multiple socioeconomic burdens and competing demands provide crucial context for understanding the challenges in accessing formal spiritual care. Economic hardships, limited access to healthcare, and other social determinants of health can impact patients' ability to prioritize and seek spiritual support services [35]. These burdens can also influence how patients perceive the importance of spiritual care, often relegating it to a secondary concern in the face of more immediate needs such as seeking the welfare of their homes and securing food. This highlights healthcare providers’ need to consider these socioeconomic factors when designing and implementing spiritual care services.

Furthermore, this study revealed that despite these socioeconomic challenges, patients strongly desire spiritual health to be addressed within their medical care. This finding is significant as it suggests that patients value integrating spiritual care into their overall treatment plan. Previous studies have shown that addressing spiritual needs can lead to better health outcomes, improved coping mechanisms, and higher patient satisfaction [31, 34]. However, physicians often face barriers to providing spiritual care, including lack of training, time constraints, and perceptions that such care falls outside their professional role [36, 37]. Meanwhile, seriously ill patients express a clear preference for physicians to inquire about their spiritual concerns and facilitate appropriate support. Notably, patients typically do not expect physicians to offer spiritual guidance directly but rather to act as spiritual care generalists—willing to listen and refer them to specialists, such as chaplains, who are trained to provide holistic spiritual care [38–41]. To meet these needs, healthcare facilities, particularly in the Deep South, should prioritize the development and implementation of spiritual care interventions that are interprofessional, accessible, relevant, and responsive to their patients’ needs. Clinician and patient feedback suggests that the SCAI framework is a promising spiritual care intervention for use in a predominately minority ambulatory population with low resources; however, they also highlighted improvements necessary to make the intervention more accessible and relevant. Participants stressed that interventions to address spiritual care must be patient-driven, compassionate, and integrated into medical care as a comprehensive offering. This approach aligns with the principles of patient-centered care, emphasizing the importance of involving patients in decision-making and tailoring care to their needs and preferences [42]. By ensuring that spiritual care interventions are compassionate and patient-driven, healthcare providers can better meet their patients’ emotional and spiritual needs, fostering a more supportive and healing environment.

This study has a few limitations worth noting. First, our findings are specific to the healthcare facility where the study was conducted and should not be generalized to broader populations. We caution against oversimplification of the minority experience, as regional and cultural differences may lead to variations in spiritual care needs and preferences. While spirituality may hold equal importance for other groups, such as Hispanic/Latino or Native American populations, its expression may differ significantly. For example, Hispanic/Latino individuals may emphasize collective faith practices rooted in familial bonds, while Native American communities might incorporate indigenous spiritual traditions [43–46]. Conversely, East Asian populations may draw on secular philosophies, such as Buddhism and Confucianism, as part of their coping strategies [47]. Additionally, while our Southern patient participants thought that clinicians should address spiritual health in clinical encounters, other groups may view such discussions as deeply private and inappropriate for the medical setting [48, 49]. Social determinates of health such as an overburdened healthcare system, competing demands, and limited financial resources impacted the prioritization of formal spiritual care services in our study population; however, other racial and ethnic minorities may face the additional barriers, such as language differences and mistrust of the medical system because of immigration status.

Second, the caregivers we approached did not consent to participate in our study. Therefore, our study lacks the perspective of family members who play a critical role in healthcare decision-making for seriously ill Black [50]. Familism significantly influences caregiving dynamics, often leading caregivers to take on extensive roles in healthcare decisions [51]. Research frequently highlights the reliance of Black caregivers on religious and spiritual coping mechanisms, underscoring the need to include their perspectives when developing formal spiritual care support [52, 53]. Furthermore, as with other racial and ethnic groups, caregiving often involves gendered dynamics, with Black women more likely than Black men to assume this role [54, 55]. These gendered differences may shape views on how formal spiritual care services should be implemented. One study, for example, found variability in how patients and caregivers perceive appropriate inclusion of religion and spirituality discussion in healthcare encounters [14].

This study advocates for the integration of spiritual care through interventions such as SCAI in healthcare facilities serving predominantly minority patients with low resources. The themes identified highlight the critical role of spirituality in patient well-being, the impact of socioeconomic burdens, and the necessity for patient-centered, compassionate care. Participants expressed a desire to have access to chaplains to address the themes identified in the SCAI framework. Expanded training and adaptation of SCAI for use in diverse populations by race and geographic location should be undertaken. Future research should also continue to explore developing, refining, and implementing spiritual care interventions, aiming to create standardized protocols that can be adapted to various healthcare settings. By addressing patients’ spiritual needs, healthcare providers can enhance overall patient care, improve health outcomes, and promote health equity in under-resourced communities.

## Supplementary Information

ESM 1 (DOCX 21 kb)

## Data Availability

Data supporting the findings of this study are available upon reasonable request from the corresponding author.

## Declarations

Ethics Approval

This study was performed with the principles of the Declaration of Helsinki. Approval was granted by the University of Alabama at Birmingham Institutional Review Board (IRB-30008321).
