# Supplementary material for: Exploring Spiritual Concerns, Needs, and Resources in Outpatient Healthcare Facilities Serving Under-Resourced Black Patients: A Qualitative Study
Source: J Racial Ethn Health Disparities. 2024 Dec 11;13(1):450–62. doi: 10.1007/s40615-024-02258-9 (PMC12795973; doi:10.1007/s40615-024-02258-9)
Supplement: Supplementary file 1 — (DOCX 21 kb) [file 40615_2024_2258_MOESM1_ESM.docx]

Supplemental Document 1. Interview Guide

| Type of Question | Patient | Caregiver | Clinicians |
| --- | --- | --- | --- |
| **Opening** | Hi [PARTICIPANT’S NAME]. Thank you so much for talking with me today. My name is [INTERVIEWER’S NAME], and I am a member of this study.  I expect that this interview will take about 45 minutes or so of your time. Is this still a good time?  We would like to ask you about any spiritual concerns and distress experienced by you or the person you care for as well as any resources for spiritual support that you would like to see provided by [THE COMMUNITY HEALTH SERVICE]. Next, we would like to ask for our input in developing a program of chaplain-led spiritual care at [THE COMMUNITY HEALTH SERVICE] For this phase of the study, we expect to enroll 6-8 African American persons with two or more chronic conditions, 6-8 family caregivers, and 6-8 primary care clinicians who work at [THE COMMUNITY HEALTH SERVICE] and [AFFLIATED UNIVERSITY].  I will be recording the interview; however, I will not share this recording with anyone besides individuals who are part of our research team at the [AFFLIATED UNIVERSITY]. I also want to emphasize that you may stop at any time and for any reason during the interview.  Do you have any questions for me before we start the interview, and the recorder is turned on?  OK, let’s get started…[*TURN RECORDER ON*] | | |
| **Introduction** | Tell me a little about yourself. How would you describe yourself to someone who doesn’t know you? | | |
|  | 1a) Very briefly and without going into detail, tell me about your illness/illnesses and the treatment you are currently undergoing. | 1b) What specific things do you do to assist your loved one from day to day? [ADDITIONAL PROMPTS: What kinds of things does this person assist you with around the house? With your medications? With your symptoms? With coordinating your medical care? With your physical mobility and hygiene? With how you are feeling and coping?] | 1c) Tell me about your clinical work.   - What population do you see? - How long have you been in practice? - What kind of patients do you provide care? |
| **Spirituality** | **Next, I would like to discuss what role religion and spirituality plays in your life/ your relationship with the person you provide support for/ the patients you provide care to.**  2. Please describe for me your religious or spiritual beliefs.   - 1. Prompts- specific faith tradition, rituals, frequency of attendance, importance…   2. What, if any, spiritual or religious activities do you take part in? By this I mean, tell me about the practices you perform as part of your beliefs. This could be praying, attending services, or performing faith practices.   3. What role, if any, does spirituality or religion play in your life as a whole? | | |
|  | 4. What role does spirituality and or religion play in living with illness/supporting someone with chronic illness/ providing care to chronically ill persons? | | |
|  | 5. Spirituality in health care.   1. What are your thoughts on clinicians asking about spirituality when coming up with care/treatment plans? 2. How much do you think clinicians should incorporate spirituality in patient care?    - 1. how do you think clinicians should communicate with the patient about religiosity and spirituality? | | |
| Perspectives on patients’ illness-related spiritual concerns and distress | 1. In your experience as a [patient/FCG/stakeholder/chaplain] describe for me the spiritual concerns that [you/your loved one/clients] have/has experienced. | | |
|  | 1. In your experience as a [patient/FCG/stakeholder/chaplain] describe for me spiritual distress that [you/your loved one/clients] have/has experienced. | | |
| Perspectives on potential desired spiritual support resources for outpatients at community safety net health center | 1. What spiritual care resources are currently being offered at the [COMMUNITY HEALTH SERVICE]? | | |
|  | 1. What spiritual care resources would you like to see offered to patients and family caregivers at [THE COMMUNITY HEALTH SERVICE]? | | |
| **Describe the SCAI Program** | 1. What is your overall impression of the SCAI program?    1. What do you like?    2. What do you think will not work well? | | |
| Intervention content | 1. What do you think about the open-ended questions on spiritual experiences the chaplain will ask to do his/her assessment? | | |
|  | 1. What do you think about list of potential spiritual care intervention that chaplain can provide the patients based on their need?    1. Should any be taken out?    2. Should any be included that are not there? | | |
|  | 13. In your opinion, what would be the best way to integrate family caregivers into this program? | | |
|  | 14. With some individuals we’ve worked with in the past, we noticed that they had difficulty understanding health information and some even had difficulty reading. This made it harder for them to participate in our programs. For these folks, how would you advise us to adapt our program to meet their needs? | | |
| Intervention format | 15. The potential intervention be three 30-minutes long sessions. What do you think about participants receiving three phone calls? What do you think about the length of these calls? | | |
| Intervention delivery | 16. This program would be conducted by a spiritual care specialist, or a chaplain, over the phone. What do you think about this approach? | | |
|  | 17. Should patients and caregivers participate in the program together, or separately? | | |
|  | 18. What other ways, besides the phone, should we consider to enhance our contact and engagement with patients and family caregivers? | | |
| **Closing thoughts** | 19. Do you have another other thoughts on this program or things you think I should know. | | |
